# Multimodal Phenomap of Stargardt Disease Integrating Structural, Psychophysical, and Electrophysiologic Measures of Retinal Degeneration

**DOI:** 10.1016/j.xops.2023.100327

**Published:** 2023-05-09

**Authors:** Mya Abousy, Bani Antonio-Aguirre, Kanza Aziz, Ming-Wen Hu, Jiang Qian, Mandeep S. Singh

**Affiliations:** 1 Wilmer Eye Institute, Johns Hopkins Hospital, Baltimore, Maryland; 2 Massachusetts Eye and Ear, Harvard Medical School, Boston, Massachusetts; 3 Department of Genetic Medicine, Johns Hopkins University, Baltimore, Maryland

**Keywords:** Inherited retinal disease, Juvenile macular dystrophy, Optical coherence tomography, Confocal scanning laser ophthalmoscopy, ABCA4

## Abstract

**Objective:**

To cluster the diverse phenotypic features of Stargardt disease (STGD) using unsupervised clustering of multimodal retinal structure and function data.

**Design:**

Retrospective cross-sectional study.

**Subjects:**

Eyes of subjects with STGD and fundus autofluorescence (FAF), OCT, electroretinography (ERG), and microperimetry (MP) data available within 1 year of the baseline evaluation.

**Methods:**

A total of 46 variables from FAF, OCT, ERG, and MP results were recorded for subjects with STGD as defined per published criteria. Factor analysis of mixed data identified the most informative variables. Unsupervised hierarchical clustering and silhouette analysis identified the optimal number of clusters to classify multimodal phenotypes.

**Main Outcome Measures:**

Phenotypic clusters of STGD subjects and the corresponding cluster features.

**Results:**

We included 52 subjects and 102 eyes with a mean visual acuity (VA) at the time of multimodal testing of 0.69 ± 0.494 logarithm of minimum angle of resolution (20/63 Snellen). We identified 4 clusters of eyes. Compared to the other clusters, cluster 1 (n = 16) included younger subjects, VA greater than that of clusters 2 and 3, normal or moderately low total macular volume (TMV), greater preservation of scotopic and photopic ERG responses and fixation stability, less atrophy, and fewer flecks. Cluster 2 (n = 49) differed from cluster 1 mainly with less atrophy and relatively stable fixation. Cluster 3 (n = 10) included older subjects than clusters 1 and 2 and showed the lowest VA, TMV, ERG responses, and fixation stability, with extensive atrophy. Cluster 4 (n = 27) showed better VA, TMV similar to clusters 1 and 2, moderate ERG activity, stable fixation, and moderate-high atrophy and flecks.

**Conclusions:**

Reflecting the phenotypic complexity of STGD, an unsupervised clustering approach incorporating phenotypic measures can be used to categorize STGD eyes into distinct clusters. The clusters exhibit differences in structural and functional measures including quantity of flecks, extent of retinal atrophy, visual fixation accuracy, and ERG responses, among other features. If novel pharmacologic, gene, or cell therapy modalities become available in the future, the multimodal phenomap approach may be useful to individualize treatment decisions, and its utility in aiding prognostication requires further evaluation.

**Financial Disclosure(s):**

Proprietary or commercial disclosure may be found after the references.

Stargardt disease (STGD) is the most common hereditary juvenile retinopathy with an estimated prevalence of 1 in 10 000.^1^, ^2^, ^3^ It is an autosomal recessive disorder caused by mutations in the *ABCA4* gene which belongs to the transmembrane ATP-binding cassette transporter proteins gene family.^1^^,^^4^ The phenotypic manifestations of STGD are diverse, and the multiple biomarkers show considerable severity range. Mutations in this gene lead to an accumulation of lipofuscin in the retinal pigment epithelium (RPE), causing RPE and photoreceptor layer atrophy.^4^, ^5^, ^6^ Lipofuscin accumulates in flecks, hallmark lesions of STGD that may be visualized biomicroscopically as white-yellow lesions in the posterior pole^2^^,^^3^ and by fundus autofluorescence (FAF) as hyperautofluorescent (AF) foci. OCT may demonstrate intraretinal or subretinal flecks in addition to foveal cavitation and outer retina thinning,^7^ and FAF often demonstrates 1 or many atrophic lesions in the macula.^2^ Lesions that spare the fovea are associated with better baseline visual acuity (VA).^8^ Patients usually present with symptoms in early childhood, but adult-onset STGD has been associated with significantly better visual prognosis.^3^^,^^9^, ^10^, ^11^ Clinical symptoms of STGD include bilateral progressive central scotoma, photophobia, and difficulty with color vision in some cases.^2^^,^^12^

The natural history of STGD is well described.^3^^,^^13^, ^14^, ^15^, ^16^, ^17^ The Natural History of the Progression of Atrophy Secondary to STGD (ProgStar) is a collection of studies that characterized longitudinal changes both prospectively and retrospectively in subjects with STGD.^3^ Recognizing the phenotypic complexity of STGD and the multitude of testing modalities used in research and clinical practice to capture structural and functional features, the ProgStar studies analyzed FAF, OCT, microperimetry (MP), VA, and demographic information to draw patterns regarding subject phenotypes. For example, FAF imaging analysis on macular atrophy lesion size and growth rate revealed that lesion growth rate depended on initial lesion size.^15^ Kuehlewein et al.^18^ identified 2 types of atrophy in STGD on FAF imaging: definitely decreased autofluorescence (DDAF) and questionably decreased autofluorescence (QDAF). Approximately 50% of subjects in the ProgStar study with genetically confirmed STGD but no atrophic lesions were found to develop a DDAF lesion within 5 years.^16^ In the fourth ProgStar report, a decrease in areas of QDAF was associated with an increase in DDAF area, suggesting that QDAF areas may transform into DDAF lesions rather than the 2 lesions being mutually exclusive entities.^17^

There are > 900 reported mutations in *ABCA4* that result in STGD which may account for the considerable phenotypic diversity.^2^^,^^19^ This genetic heterogeneity makes classifying patients based on phenotype, also known as phenomapping, a complicated task.^20^ “Phenomapping” is a term that first gained popularity in the realm of cardiology, where Shah et al.^21^ characterized subjects with heart failure based on a variety of clinical markers. Through machine learning, specifically unsupervised and supervised clustering, Shah and colleagues identified 3 categories of subjects that comprised a unique set of phenotypic markers for each category that held potential prognostic value, could aid in treatment planning, and could also support subject selection for clinical trials. In other fields, including cardiology, risk stratification and clustering of heterogenous disease phenotypes have allowed physicians to determine when to begin certain medications using risk calculators.^22^^,^^23^ Our project is motivated by the fact that this approach may be similarly useful for STGD. To date, although multiple testing modalities have been used to characterize phenotypic severity in STGD, there is no statistically validated classification algorithm that incorporates a variety of imaging and retinal function test data to create a comprehensive “phenomap,” or a description of phenotypic diversity and range, of STGD. Applying phenomapping techniques to STGD may lead to the definition of therapeutically homogeneous subclasses of patients who may benefit from certain investigational interventions, such as stem cell, pharmaceutical, or gene therapy. In addition, phenomapping may aid in risk stratification and prognosis of the disease.^22^^,^^23^ This approach to more personalized medicine may revolutionize the clinical management of STGD.

There have been prior attempts to classify STGD phenotypes that do not use clustering methods: Lois et al. established 3 phenotypes of STGD based on electroretinogram (ERG) data, and Fujinami et al. used OCT, FAF, ERG, and VA to categorize subjects based on severity of phenotype.^24^^,^^25^ Arrigo et al.^26^ analyzed several OCT parameters of subjects with STGD and identified vessel tortuosity as a distinctive variable that could separate subjects based on VA. These classification systems are highly informative and show that there are many testing modalities that can capture the phenotypic diversity of STGD. Further, the published classifications provide broad support for the idea that integrating data from a greater number of test modalities and employing machine learning via unsupervised clustering, as we propose here, may yield a more powerful approach to categorize phenotypes. To address our goal to evaluate a comprehensive phenotype classification approach for STGD, we aimed to identify categories of subjects with STGD based on a variety of imaging and retinal function modalities. We used unsupervised learning algorithms to generate a data-driven grouping using relevant clinical, structural, and functional variables. To enable our work, we leveraged an existing database of subjects with STGD at the Johns Hopkins Genetic Eye Diseases Center.

## Methods

### Study Design and Participants

This retrospective cross-sectional study identified eligible subjects at the Wilmer Eye Institute at the Johns Hopkins Hospital from January 2008 to February 2022. The institutional review board of Johns Hopkins University approved this study. Study activities adhered to the guidelines of the Declaration of Helsinki. This study is a retrospective cross-sectional study that uses de-identified patient information and only involves chart review. Consent was not required for this study, and this was approved by the institutional review board. Subjects were included if they had either 2 disease-causing mutations in *ABCA4* or 1 disease-causing mutation and a typical STGD phenotype with flecks at the level of the RPE, per the published ProgStar criteria.^15^ Exclusion criteria included lack of all required imaging and retina function testing available within 1 year of the baseline evaluation. Prespecified variables (Table 1) were extracted from electronic medical records, including clinical (age, VA), structural (FAF, OCT), and functional (ERG, MP) retina parameters. Visual acuity was recorded as a Snellen fraction which was then converted to logarithm of the minimum angle of resolution (logMAR) for the analysis. Fundus autofluorescence, OCT, ERG, and MP tests were graded by a trained researcher (M.A.) and verified with a retinal specialist (M.S.S.). Graders were not masked to patient indentification, as for this project retrospective clinical images were reviewed on the commercially available Heidelberg software.

**Table 1:** Description, Categorization, and Source Modality of Extracted Variables

Variable	Options	Type of Variable	Modality
Age		Continuous	
logMAR visual acuity		Continuous	
Background heterogeneity	Homogeneous without flecks beyond arcades; Homogeneous with flecks beyond arcades; Heterogenous with flecks beyond arcades	Ordinal	FAF
Fujinami classification	1 low signal, homogeneous background; 1 low signal, heterogeneous background; Multiple low signals, heterogeneous background	Ordinal	FAF
Flecks present overall?	0; 1	Binary	FAF
Total number of flecks	0–50; 50–100; 100+	Ordinal	FAF
Presence of flecks in zone 1	0; 1	Binary	FAF
Number of flecks in zone 1	0–50; 50–100; 100+	Ordinal	FAF
Presence of flecks in zone 2	0; 1	Binary	FAF
Number of flecks in zone 2	0–50; 50–100; 100+	Ordinal	FAF
Presence of flecks in zone 3	0; 1	Binary	FAF
Number of flecks in zone 3	0–50; 50–100; 100+	Ordinal	FAF
Lesion edge hyperautofluorescent?	0; 1	Binary	FAF
Presence of DDAF in zone 1	0; 1	Binary	FAF
Presence of DDAF in zone 2	0; 1	Binary	FAF
Presence of DDAF in zone 3	0; 1	Binary	FAF
Presence of QDAF in zone 1	0; 1	Binary	FAF
Presence of QDAF in zone 2	0; 1	Binary	FAF
Presence of QDAF in zone 3	0; 1	Binary	FAF
Atrophic lesion(s) spare(s) fovea?	0; 1	Binary	FAF
Predominant lesion in zone 1	No lesions; QDAF; DDAF; Flecks	Nominal	FAF
Predominant lesion in zone 2	No lesions; QDAF; DDAF; Flecks	Nominal	FAF
Predominant lesion in zone 3	No lesions; QDAF; DDAF; Flecks	Nominal	FAF
Atrophy beyond arcades?	0; 1	Binary	FAF
Tubulation present?	0; 1	Binary	OCT
Fovea gap present?	0; 1	Binary	OCT
Intraretinal fluid present?	0; 1	Binary	OCT
Outer retinal atrophy?	0; 1	Binary	OCT
Total macular volume		Continuous	OCT
Center subfield thickness		Discrete	OCT
Center point thickness		Discrete	OCT
Center subfield volume		Continuous	OCT
Central macular minimum		Discrete	OCT
Central macular maximum		Discrete	OCT
DA 0.01 a-wave amplitude		Discrete	ERG
DA 3.0 a-wave amplitude		Discrete	ERG
DA 3.0 b-wave amplitude		Discrete	ERG
LA 3.0 a-wave amplitude		Discrete	ERG
LA 30Hz amplitude		Discrete	ERG
DA 3.0 a-wave latency		Discrete	ERG
DA 3.0 b-wave latency		Discrete	ERG
LA 3.0 a-wave latency		Discrete	ERG
LA 30Hz latency		Discrete	ERG
% Fixation points within 1° from fovea		Discrete	MP
% Fixation points within 2° from fovea		Discrete	MP
Fixation stability	Unstable; relatively unstable; stable	Ordinal	MP

0 = no; 1 = yes; DA = dark adapted; DDAF = definitely decreased autofluorescence; ERG = electroretinogram; FAF = fundus autofluorescence; LA = light adapted; logMAR = logarithm of the minimal angle of resolution; MP = microperimetry; QDAF = questionably decreased autofluorescence.

### FAF Analysis

We studied 22 variables collected from the FAF images, which were taken with a Spectralis HRA + OCT camera (Heidelberg Engineering). Background heterogeneity and presence of flecks beyond the arcade was assessed as described in previous literature.^14^^,^^27^^,^^28^ The scale established by Fujinami et al.^28^ was additionally used to incorporate the number of atrophic lesions and background heterogeneity. Briefly, the Fujinami system classified subjects as type 1 if the FAF showed a homogeneous background with 1 low AF foveal lesion, type 2 if there was a heterogeneous background with 1 low AF lesion, and type 3 if there was a heterogeneous background and multiple low AF lesions.^28^

**Figure 1:**
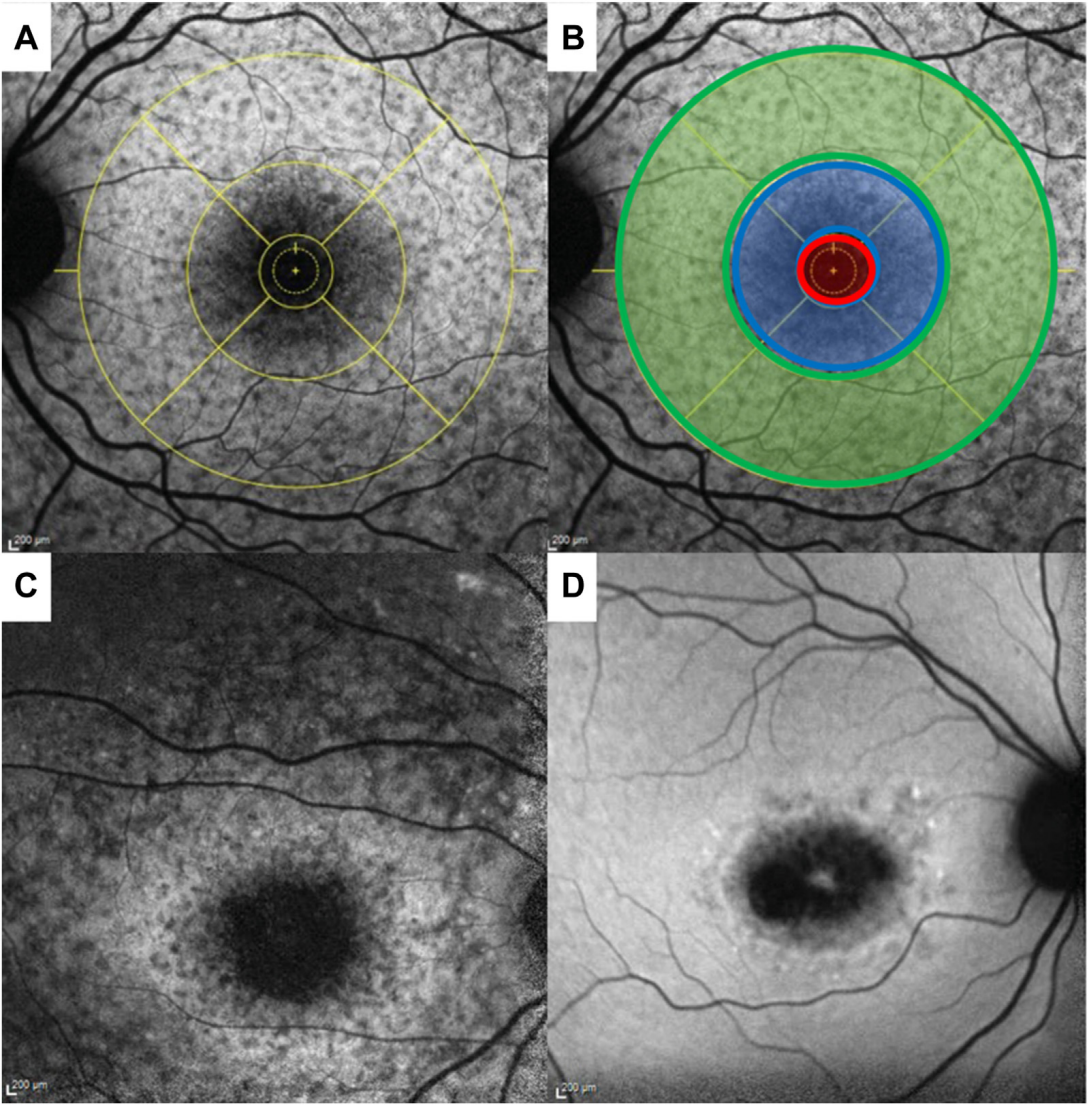
**A,** Left eye (OS) fundus autofluorescence image of patient with Stargardt disease with ETDRS grid placed at approximated foveola location. **B,** zones 1, 2, and 3 were identified by the red, blue, and green circles, respectively. Characteristics of each zone were documented for each OS fundus autofluorescence scan. **C,** hyperautofluorescent ring surrounding region of definitely decreased autofluorescence (DDAF). **D,** fovea-sparing lesion. There is a small, circular region at the fovea with no atrophic lesions surrounded by a combination of DDAF and questionably decreased autofluorescence for 180°.

The presence of flecks in any zone, as well as presence of flecks beyond the arcade and in zones 1-3 (per ETDRS) grid, as shown in Fig 1A, B), were documented. The number of total flecks and flecks in each zone were further documented. Hyperautofluorescent rings were identified (Fig 1C). Definitely decreased autofluorescence and QDAF were identified based on prior classifications by Kuehlewein et al.^18^ The former referred to lesions that were ≥ 90% as dark as the optic nerve or vessels, which served as a reference point, while the latter referred to lesions that were between 50% and 90% as dark. The presence of DDAF and QDAF in zones 1–3 was documented along with the type of lesion with the greatest surface extent. Lesions that spared the fovea (either DDAF or QDAF lesions) were defined according to published criteria (Fig 1D).^29^ Predominant lesions (DDAF, QDAF, flecks, no lesions) in each zone were documented as well as the presence of atrophy beyond the arcades.

### OCT Analysis

**Figure 2:**
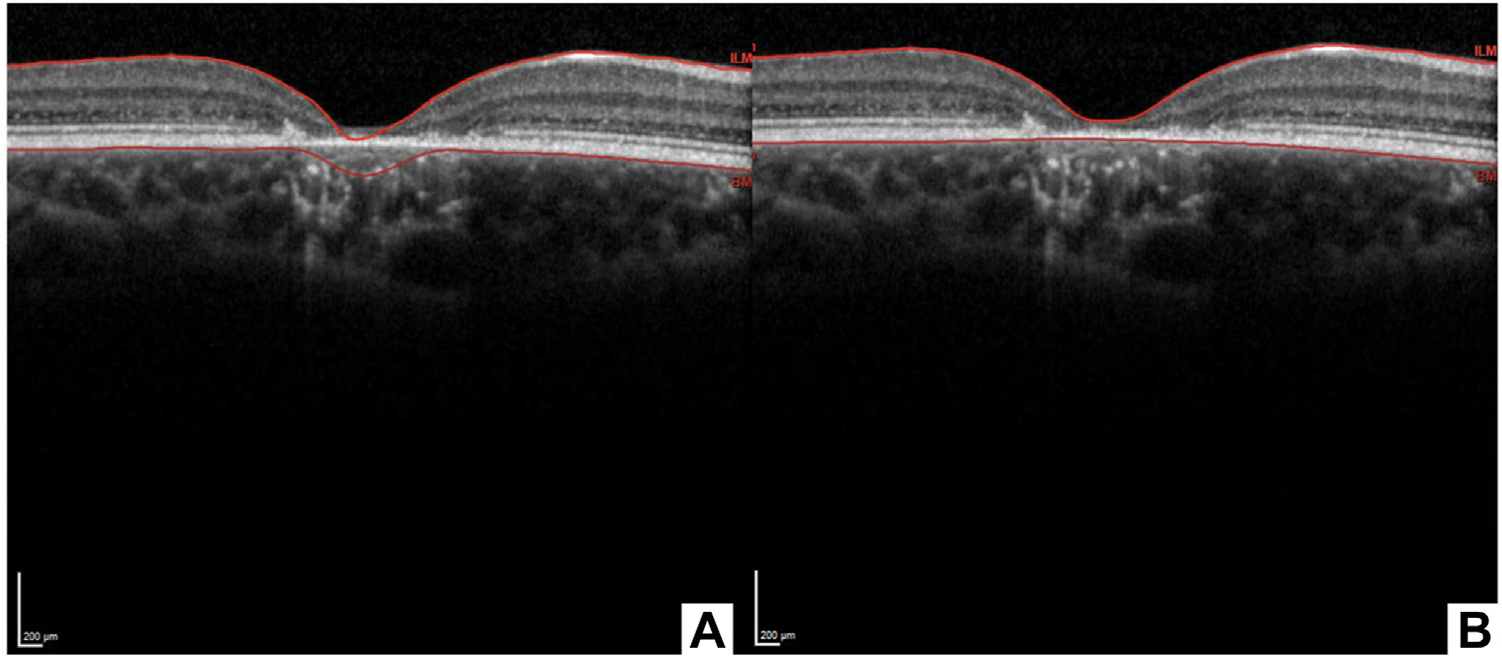
Automated segmentation of internal limiting membrane and Bruch's membrane (**A**) by the Heyex program and (**B**) corrected manual segmentation performed prior to documenting retinal thicknesses.

We selected 10 OCT variables for the analysis, retrieved from OCT images taken with a Spectralis HRA + OCT camera (Heidelberg Engineering). We assessed the presence of outer retinal tubulations, fovea gaps, intraretinal fluid, thinning/thickening of the RPE, and outer retinal atrophy in each OCT image. Macular thickness measurements were recorded using the Thickness Map feature on the Heidelberg Eye Explorer (Heidelberg Engineering). Before these measurements were recorded, the thickness profile was assessed to ensure that the inner limiting membrane and Bruch’s membrane layers were correctly segmented. Several OCTs required manual correction of these layers (Fig 2). The total macular volume (TMV), center subfield thickness (CST), center point thickness, center subfield volume, central macular minimum, and central macular maximum were all recorded.

### ERG Data

Full-field ERGs were performed using the ColorDome ERG (Diagnosys) according to The International Society for Clinical Electrophysiology of Vision standard of 2022.^30^ We included 9 full-field ERG data points: dark adapted (DA) 0.01 a-wave amplitude, DA 3.0 a-wave amplitude and latency, DA 3.0 b-wave amplitude and latency, light adapted (LA) 3.0 a-wave amplitude and latency, and LA 30Hz amplitude and latency.

### MP Data

Three MP variables were recorded: percentage of fixation points located within 1 and 2 degrees from the fovea and fixation stability grade. The MP data were collected either from Nidek MP-1 (Nidek) or Macular Integrity Assessment (CenterVue) machines. Due to differences in testing protocol (MP-1 vs. Macular Integrity Assessment), MP mean sensitivity values were not collected in this study.^31^^,^^32^

### Statistical Analysis

We conducted Factor Analysis of Mixed Data (FAMD) to identify informative variables based on the first 3 principal component analysis (PCA) components (PCA1: 19.28%, PCA2: 12.35%, PCA3: 10.30%) in our data. We calculated the contribution of each variable in these 3 components respectively and used the average contribution of the total 46 variables as the threshold to select 36 variables (i.e., greater than the average). We further conducted correlation analysis to remove redundant variables. Pearson and Cramer’s V were used for calculating pairwise correlations in numerical and categorial variables, respectively. Following another FAMD analysis with the 17 remaining nonredundant variables, we applied unsupervised hierarchical clustering to the updated first 3 components (PCA1: 24.29%, PCA2: 13.54%, PCA3: 9.92%) to identify phenotypic clusters. To better visualize the cluster distribution, we used Uniform Manifold Approximation and Projection, a nonlinear dimensionality reduction technique. For testing statistical significance of the 17 variables among the 4 identified clusters, we used Fisher exact test in categorical variables and the Wilcoxon test in numerical variables. *P* values were adjusted by Holm’s method for pairwise comparisons. Results were considered significant at α < 0.05.

## Results

### Study Sample

**Figure 3:**
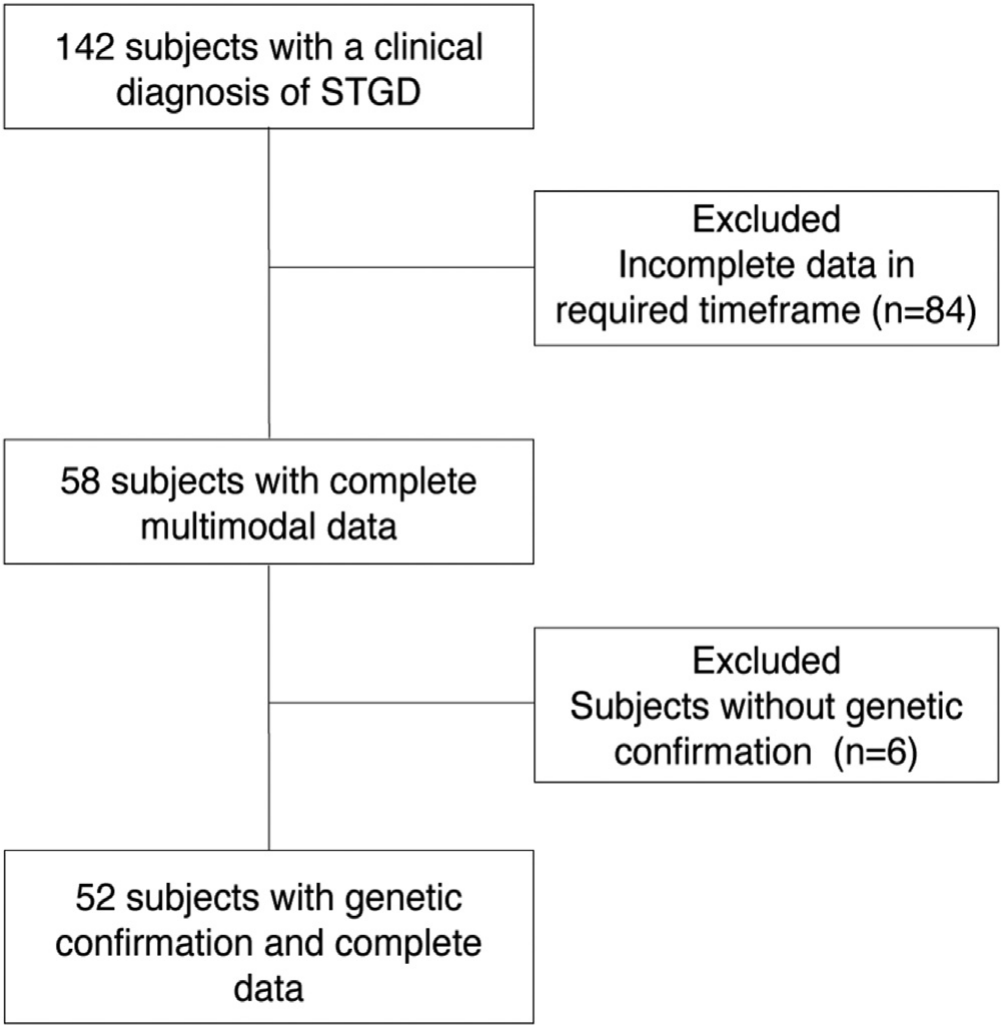
Flowchart of patient population selection. STGD = Stargardt disease.

**Figure 4:**
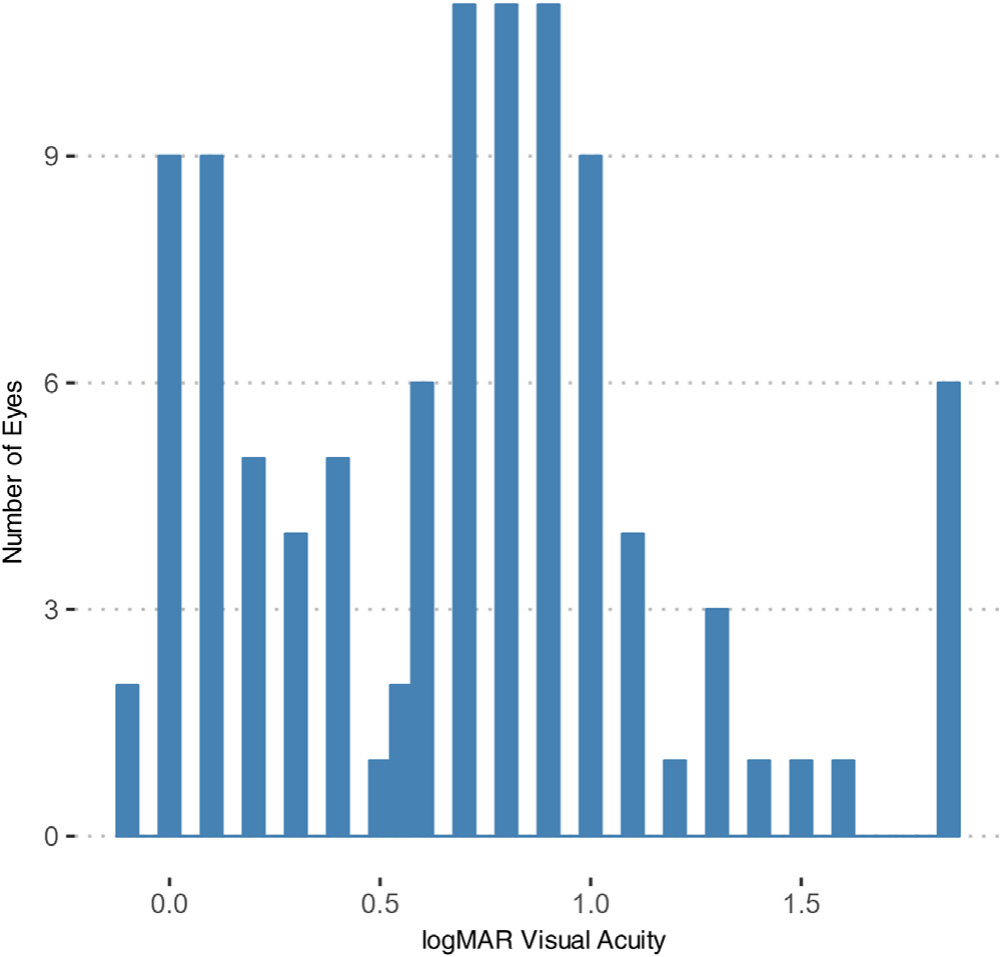
Histogram of logarithm of the minimum angle of resolution (logMAR) visual acuity.

Of the 142 subjects screened with suspected STGD based on clinical features, 90 subjects were excluded due to incomplete phenotypic records within the required timeframe (Fig 3). Thus, we included 102 eyes from 52 subjects. Of the 52 subjects, 3 biological sibling pairs were identified: (subject identifier 9 and 35, 16 and 41, 2 and 59). All other subjects were not biologically related. 47.1% of the 52 subjects were female. The mean age of subjects was 34.5 ± 16.5 years at the time of evaluation. Most subjects had 2 *ABCA4* disease-causing mutations (82%) and the remainder had 1 mutation with a typical phenotype. The mean VA was 0.69 ± 0.494 logMAR (20/63 Snellen) (Table 2 and Fig 4). The most frequent mutation was c.5461-10T>C (9.57%) (Table 3).

**Table 2:** Sample Characteristics (n = 102 Eyes/52 Patients)

Characteristics	N. of Eyes (%)	Mean ± SD	Range
Age∗		34.5 ± 16.5	10.7, 76.5
Visual acuity (logMAR)		0.691 ± 0.494	−0.0970, 1.85
Visual acuity (decimal)		0.345 ± 0.333	0.0140, 1.25
Female†	48 (47.1)		
Genetic Diagnosis			
1 *ABCA4* mutation.with typical phenotype‡	18 (17.6)		
2 *ABCA4* mutations§	84 (82.4)		

logMAR = logarithm of the minimal angle of resolution; N = number; SD = standard deviation.

Two patients only had data available for 1 eye.

∗ Age recorded at date of fundus autofluorescence testing.

† 25 female patients.

‡ 9 patients with 1 *ABCA4* mutation with the typical phenotype.

§ 43 patients with 2 *ABCA4* mutations, and a total of 52 patients.

**Table 3:** Genetic Mutations on the *ABCA4* Gene for Each Subject

Cluster	Subject/Eyes	Mutation 1	Mutation 2	Mutation 3	Most Common
1					6320G>A
	2/OS	6449G>A	6320G>A		
	5/OS	4919G>A	3386G>T		
	11/OU	1A>G	5882G>		
	16/OU	2588G>C	4253+5G>A		
	31/OU	6320G>A	2966T>C		
	35/OU	5461-10T>C	5714 + 5G>A		
	39/OU	2966C>T	6320G>A		
	58/OU	5461-10T>C	4253+43G>A		
	59/OU	6449G>A	6320G>A		
2					5461-10T>C
	3/OD	1819G>A	1244A>G		
	4/OU	4918C>T	6320G>A		
	5/OD	4919G>A	3386G>T		
	6/OU	2966C>T	4634 + 1del		
	7/OU	2034G>T	6089G>A		
	9/OU	5461-10T>C	5714+5G>A		
	10/OU	5882G>A	5881G>A	1609C>T	
	13/OU	5882G>A	5527C>G		
	15/OD	5196 + 3_5196 + 6del	5113C>T		
	19/OU	5461-10T>C	203C>G		
	24/OU	6191C>T			
	26/OU	5461-10T>C			
	27/OU	161G>A	2588G>C		
	29/OU	2588G>C	6098T>G		
	30/OU	5461-10T>C			
	32/OU	2453G>A	4532C>A		
	33/OU	3322C>T	6079C>T		
	38/OU	5882G>A	179C>T		
	41/OU	4253 + 5G>A	2588G>C		
	44/OU	5882G>A	3758C>T		
	45/OU	5461-10T>C	1937-1G>A		
	47/OU	2588G>C			
	49/OU	2588G>C	4947delC	1715G>A	
	54/OS	6320G>A	2980_2987del		
	55/OD	2041C>T	5603A>T		
	56/OU	1927G>A			
	57/OU	5196 + 1137G>A			
3					2588G>C
	3/OS	1819G>A	1244A>G		
	8/OU	2588G>C	4139C>T		
	12/OU	2588G>C	5714 + 5G>A		
	17/OU	4139C>T	4594G>A		
	25/OD	4469G>A	5537T>C		
	53/OU	1933G>A	1822T>A		
4					5461-10T>C
	14/OU	5461-10T>C			
	15/OS	5196 + 3_5196 + 6del	5113C>T		
	18/OU	1161G>A	5603A>T		
	20/OU	5882G>A			
	22/OU	6089G>A	5461-10T>C		
	25/OS	4469G>A	5537T>C		
	28/OU	6718A>G			
	34/OS	4363T>C	4253+43G>A		
	36/OU	70C>T	4594G>A		
	40/OU	428C>T	1928T>G		
	42/OU	1927G>A	3259G>A		
	43/OU	283T>C			
	51/OU	5461-10T>C			
	54/OD	6320G>A	2980_2987del		
	55/OS	2041C>T			
	60/OU	3364G>A	4685T>C		

OD = right eye; OS = left eye; OU = both eyes.

### Variable Selection

**Figure 5:**
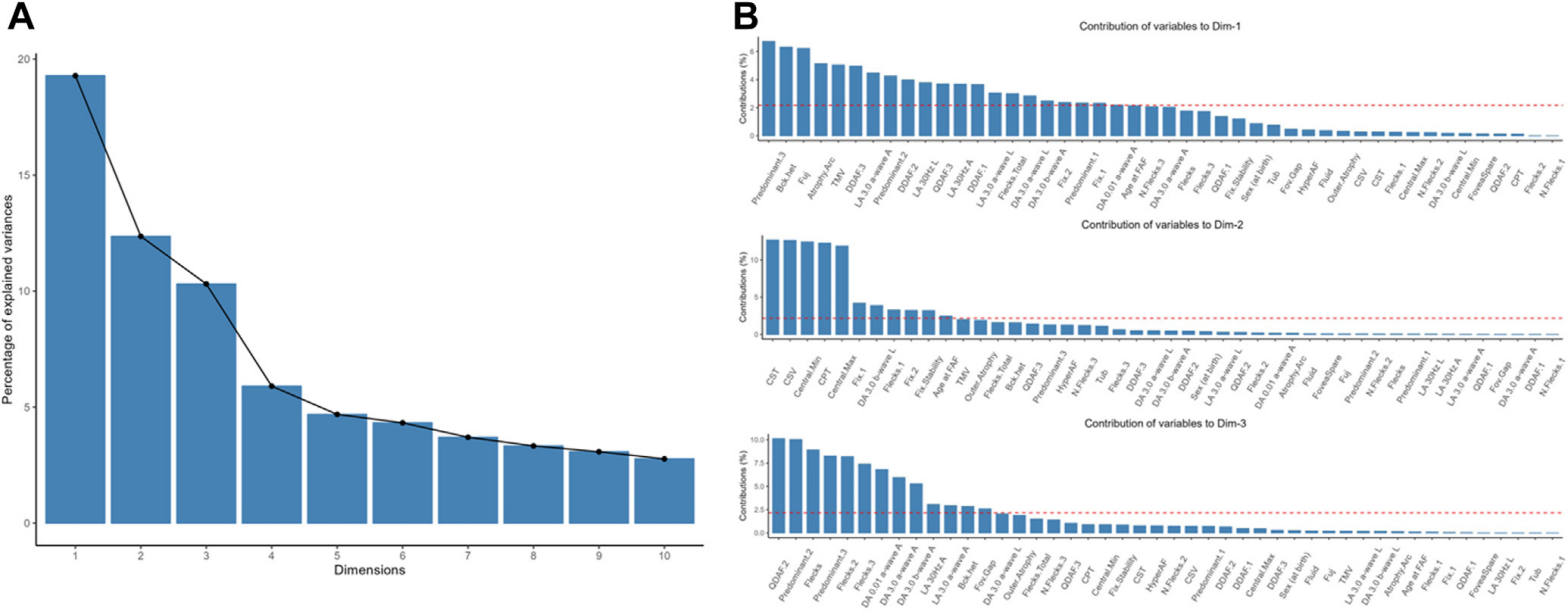
Percentage of explained variances based on various dimensions incorporating all 46 variables (**A**). The first 3 components of the principal components analysis accounted for 19.28%, 12.35%, and 10.30% of the explained variance ratio. The first 3 dimensions were used as principal components to extract the most contributory variables (**B**). AF = autofluorescent; CPT = central point thickness; CST = center subfield thickness; CSV = central subfield volume; DA = dark adapted; DDAF = definitely decreased autofluorescence; FAF = fundus autofluorescence; LA = light adapted; QDAF = questionably decreased autofluorescence; TMV = total macular volume.

**Figure 6:**
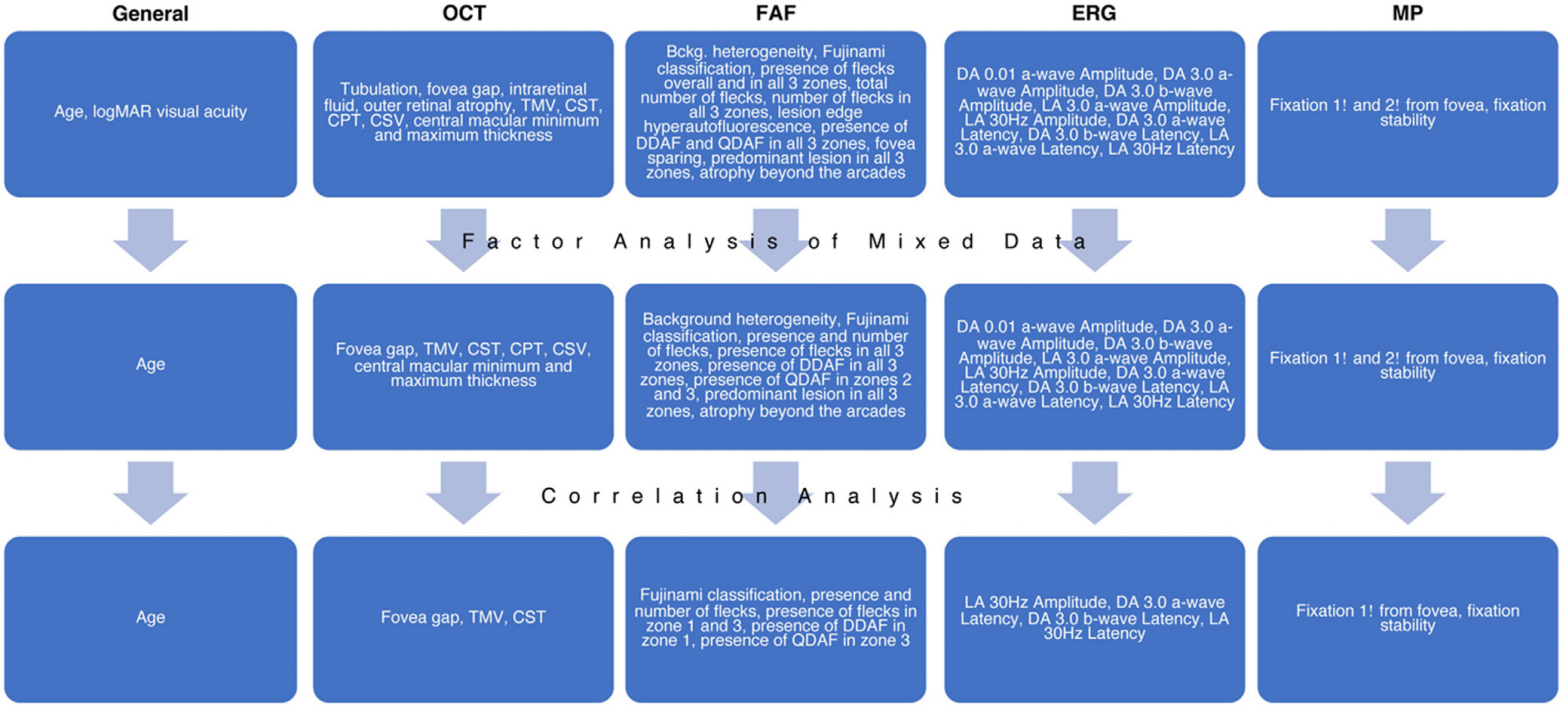
Variable selection process. The top row of variables lists all variables collected for all eyes. The second row lists the variables that remained after the factor analysis of mixed data. The third row lists the nonredundant variables after correlation analysis. Bckg. = background; CPT = central point thickness; CST = central subfield thickness; CSV = central subfield volume; DA = dark adapted; DDAF = definitely decrease autofluorescence; ERG = electroretinography; FAF = fundus autofluorescence; LA = light adapted; logMAR = logarithm of the minimum angle of resolution; MP = microperimetry; QDAF = questionably decreased autofluorescence; TMV = total macular volume.

**Figure 7:**
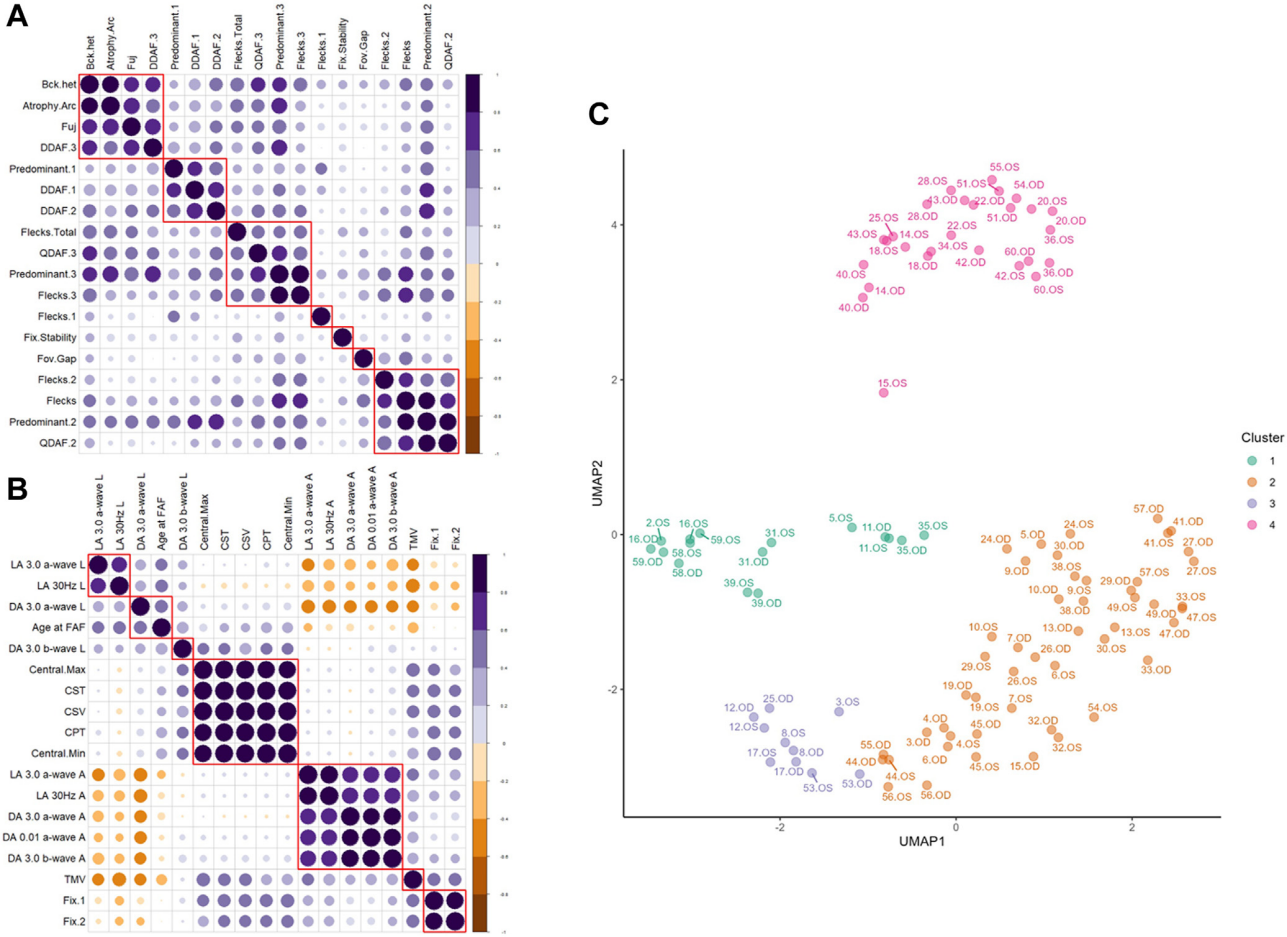
Graphical representation of correlations between the 18 categorical (**A**) and 18 continuous (**B**) variables extracted from factor analysis of mixed data. The highly correlated clusters in (**A**) and (**B**) were used to eliminate 19 variables for a total of 17 variables in the final analysis. Purple dots signify positive correlations while orange dots are negative correlations. Silhouette analysis was used to arrive at 4 final clusters (Uniform Manifold Approximation and Projection [UMAP] representation) (**C**). Atrophy.Arc = atrophy beyond the arcades; Bck.het = background heterogeneity; Central.Max = central macular thickness maximum; Central.Min = central macular thickness minimum; CPT = central point thickness; CST = central subfield thickness; CSV = central subfield volume; DA 3.0 a-wave A = Dark adapted 3.0 a-wave amplitude; DA 0.01 a-wave A = Dark adapted 0.01 a-wave amplitude; DA 3.0 b-wave A = Dark adapted 3.0 b-wave amplitude; DA 3.0 a-wave L = Dark adapted 3.0 a-wave latency; DA 3.0 b-wave L = Dark adapted 3.0 b-wave latency; DDAF.1 = presence of DDAF in zone 1; DDAF.2 = presence of DDAF in zone 2; DDAF.3 = presence of DDAF in zone 3; FAF = fundus autofluorescence; Fix.1 = percentage of fixation points 1 degree from the fovea; Fix.2 = percentage of fixation points 2 degrees from the fovea; Fix.Stability = fixation stability; Flecks = presence of flecks overall; Flecks.1 = presence of flecks in zone 1; Flecks.2 = presence of flecks in zone 2; Flecks.3 = presence of flecks in zone 3; Flecks.Total = number of flecks in entire FAF; Fov.Gap = presence of foveal gap; Fuj = Fujinami classification; LA 3.0 a-wave A = Light adapted 3.0 a-wave amplitude; LA 30Hz A = Light adapted 30Hz amplitude; LA 3.0 a-wave L = Light adapted 3.0 a-wave latency; LA 30Hz L = Ligth adapted 30Hz latency; Predominant.1 = predominant lesion in zone 1; Predominant.2 = predominant lesion in zone 2; Predominant.3 = predominant lesion in zone 3; QDAF.2 = presence of QDAF in zone 2; QDAF.3 = presence of QDAF in zone 3; TMV = total macular volume.

From the 46 variables initially collected, we selected a limited dataset of informative variables using statistical approaches and clinical judgment to arrive at 17 nonredundant variables (Fig 5, Figs 6 and 7).

### Phenotypic Clusters

**Figure 8:**
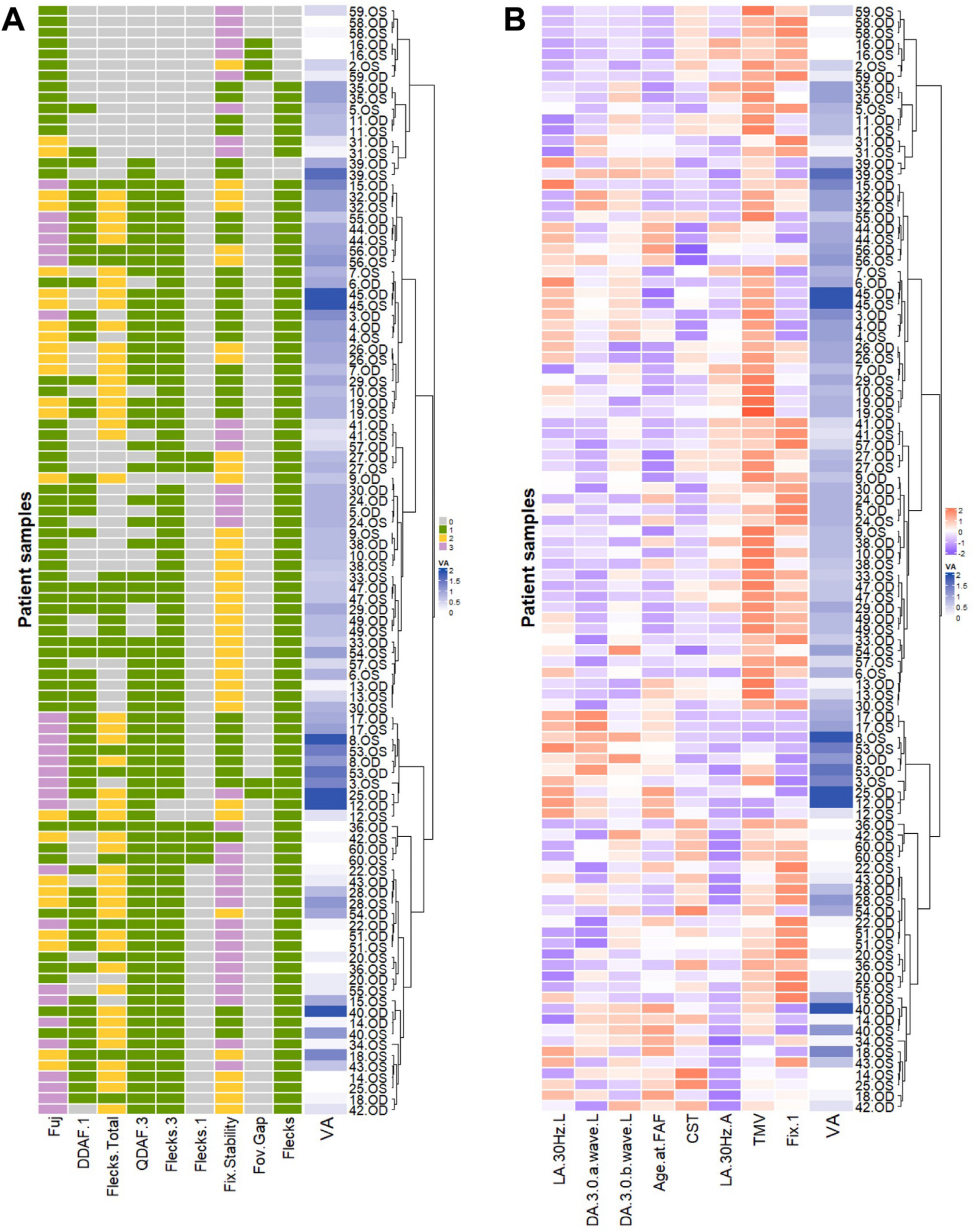
Heatmaps of categorical variables using factor analysis of mixed data (**A**) and continuous variables using hierarchical clustering (**B**) of the 17 variables for all 102 eyes. The columns represent the different variables, while the rows represent the 102 eyes. In heatmap (**A**), values are assigned based on the legend in Table 1. In heatmap (**B**), values in red are greater than the sample mean, while values in blue are less than the mean. For both heatmaps, visual acuity (VA) is represented on a logarithm of the minimum angle of resolution scale with darker values representing worse VA and lighter values representing better visual acuity. Age.at.FAF = age at FAF; CST = central subfield thickness; DA.3.0.a.wave.L = Dark adapted 3.0 a-wave latency; DA.3.0.b.wave.L = Dark adapted 3.0 b-wave latency; DDAF = definitely decrease autofluorescence; DDAF.1 = presence of DDAF in zone 1; Fix.1 = percentage of fixation points 1 degree from the fovea; Flecks = presence of flecks overall; Flecks.1 = presence of flecks in zone 1; Flecks.3 = presence of flecks in zone 3; Flecks.Total = number of flecks in entire FAF; Fov.Gap = presence of foveal gap; Fuj = Fujinami classification; LA.30Hz.A = Light adapted 30Hz amplitude; LA.30Hz.L = Light adapted 30Hz latency; OD = right eye; OS = left eye; QDAF = questionably decreased autofluorescence; QDAF.3 = presence of QDAF in zone 3; TMV = total macular volume.

**Figure 9:**
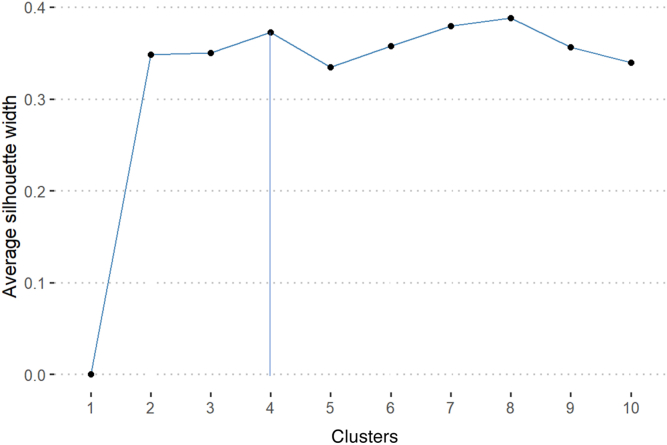
Graphical representation of the average silhouette width based on the number of clusters using the silhouette method. The optimal number of clusters was 8 and the second-most optimal number was 4.

The 17 nonredundant variables were analyzed using hierarchical clustering to generate a heatmap showing the dynamic range of all ocular phenotypes (Fig 8). The 2 eyes of each subject were clustered separately. While silhouette analysis revealed that the optimal number of phenotypic clusters was 8, the second most optimal number of clusters was 4. We decided to use the 4 phenotypic clusters for clinical practicality and to maintain a stable cluster size (Fig 9).

**Figure 10:**
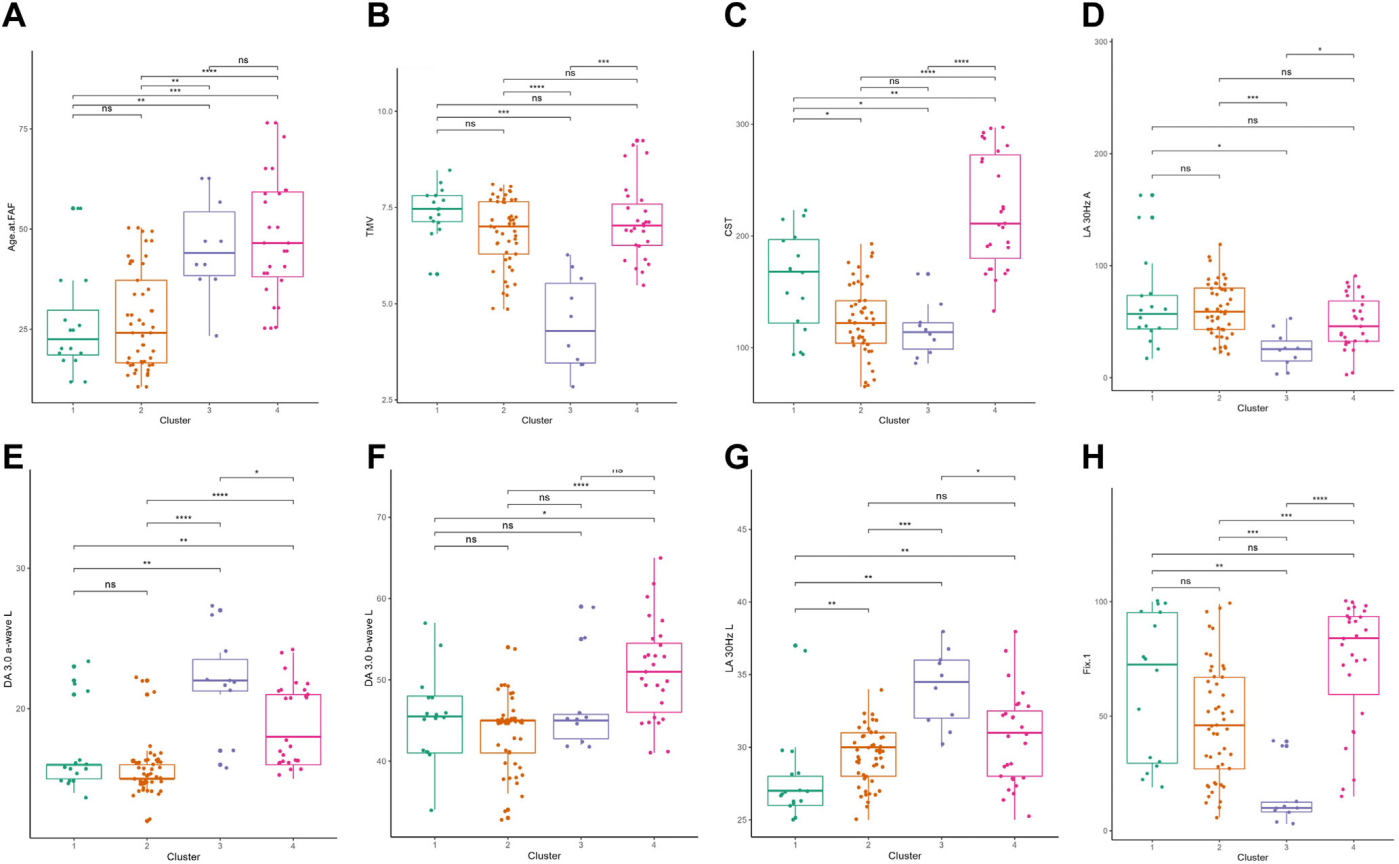
Boxplots of continuous variables based on phenotypic cluster. **A,** age at fundus autofluorescence (FAF) imaging. **B,** total macular volume (TMV). **C,** central subfield thickness (CST). **D,** light adapted (LA) 30Hz Amplitude. **E,** dark adapted (DA) 3.0 a-wave Latency. **F,** DA 3.0 b-wave Latency. **G,** LA 30Hz Latency. **H,** percentage of fixation points 1 degree from fovea. ns = not significant (*P* > 0.05). ∗*P* < 0.05, ∗∗*P* < 0.01, ∗∗∗*P* < 0.001, ∗∗∗∗*P* < 0.0001.

**Figure 11:**
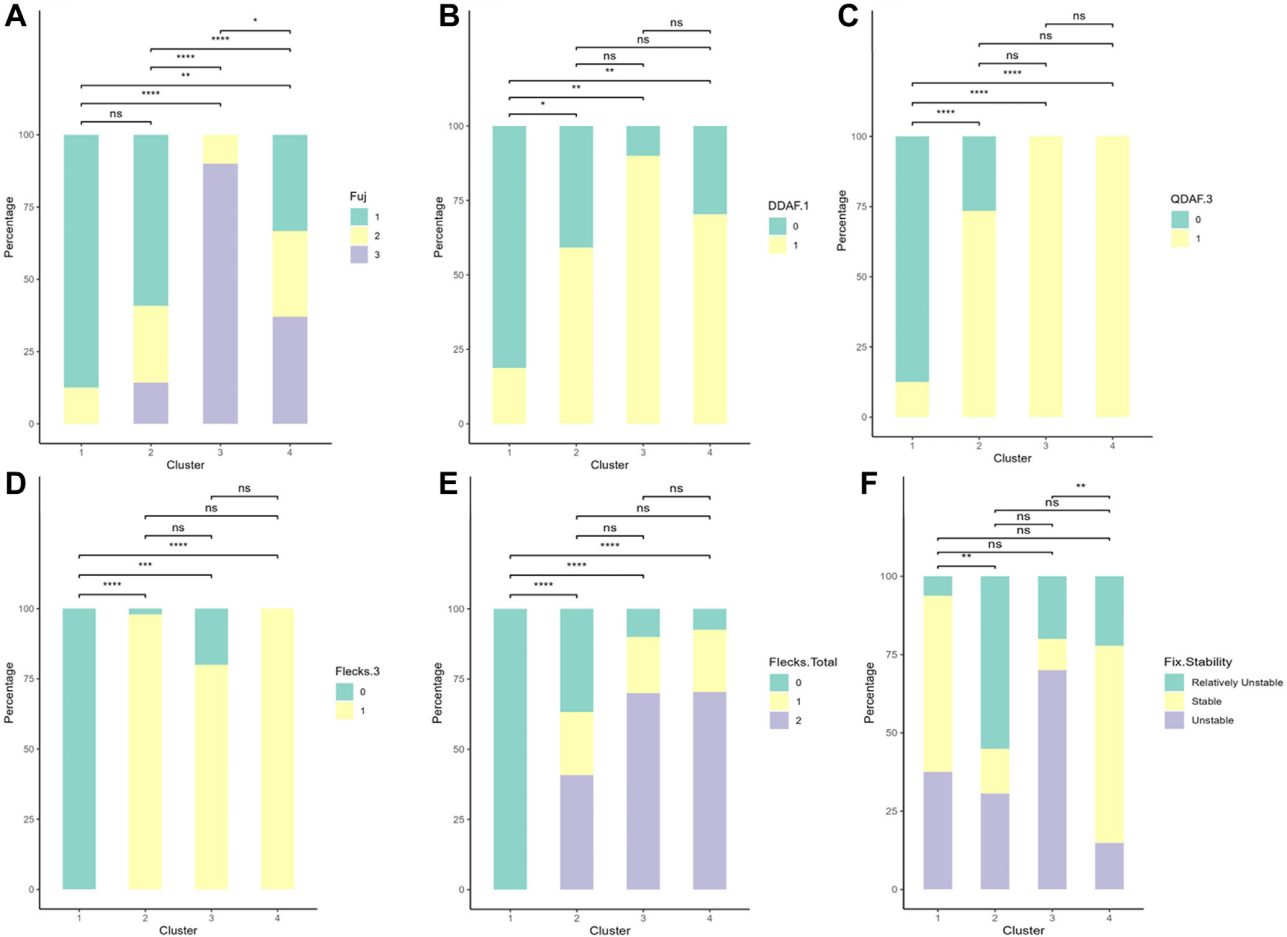
Bar graphs of percentage of eyes with each categorical variable. Each bar represents a different cluster, while the different colors represent the various categorical options for each variable. **A,** Fujinami score. **B,** presence of definitely decreased autofluorescence (DDAF) in zone 1. **C,** presence of questionably decreased autofluorescence (QDAF) in zone 3. **D,** presence of flecks in zone 3. **E,** total number of flecks in entire FAF. **F,** fixation stability. ns = not significant (*P* > 0.05). ∗*P* < 0.05, ∗∗*P* < 0.01, ∗∗∗*P* < 0.001, ∗∗∗∗*P* < 0.0001.

**Figure 12:**
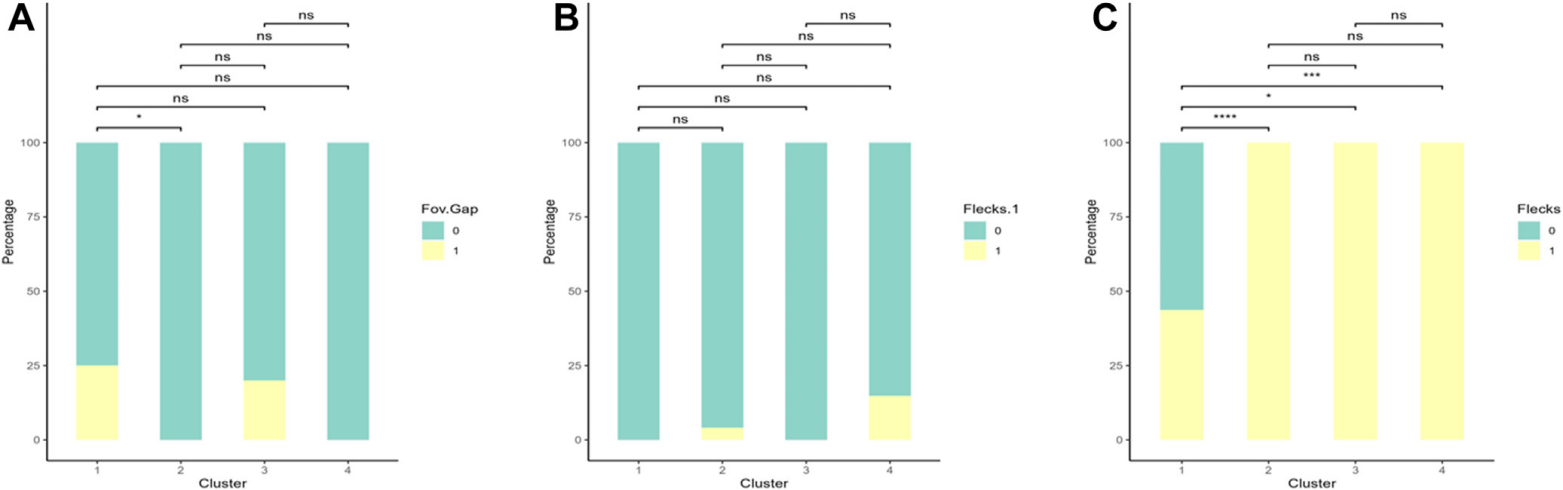
Bar graphs of percentage of eyes with each categorical variable based on cluster. Each bar graph represents a different cluster, while the different colors represent the various categorical options for each variable. **A,** presence of a fovea gap on OCT. **B,** presence of flecks in zone 1. **C,** presence of flecks in overall fundus autofluorescence. ns = not significant (*P* > 0.05). ∗*P* < 0.05, ∗∗*P* < 0.01, ∗∗∗*P* < 0.001, ∗∗∗∗*P* < 0.0001.

**Figure 13:**
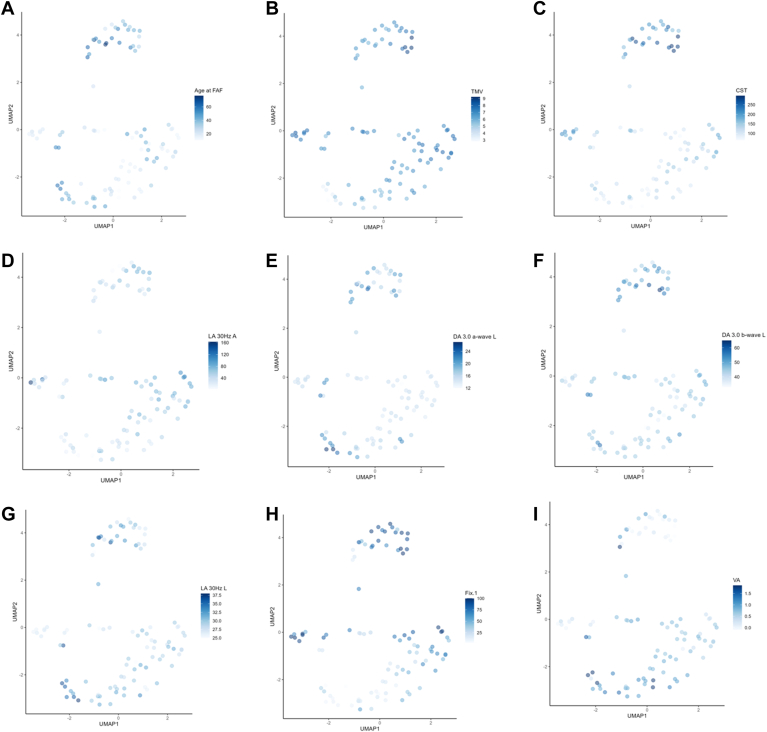
Uniform Manifold Approximation and Projection (UMAP) representation of continuous variables based on clusters. **A,** age at fundus autofluorescence (FAF) imaging. **B,** total macular volume (TMV). **C,** central subfield thickness (CST). **D,** light adapted (LA) 30Hz Amplitude. **E,** dark adapted (DA) 3.0 a -wave Latency. **F,** DA 3.0 b -wave Latency. **G,** LA 30Hz Latency. **H,** pFcoercentage of fixation points one degree from fovea. **I,** logarithm of the minimum angle of resolution visual acuity (VA).

**Figure 14:**
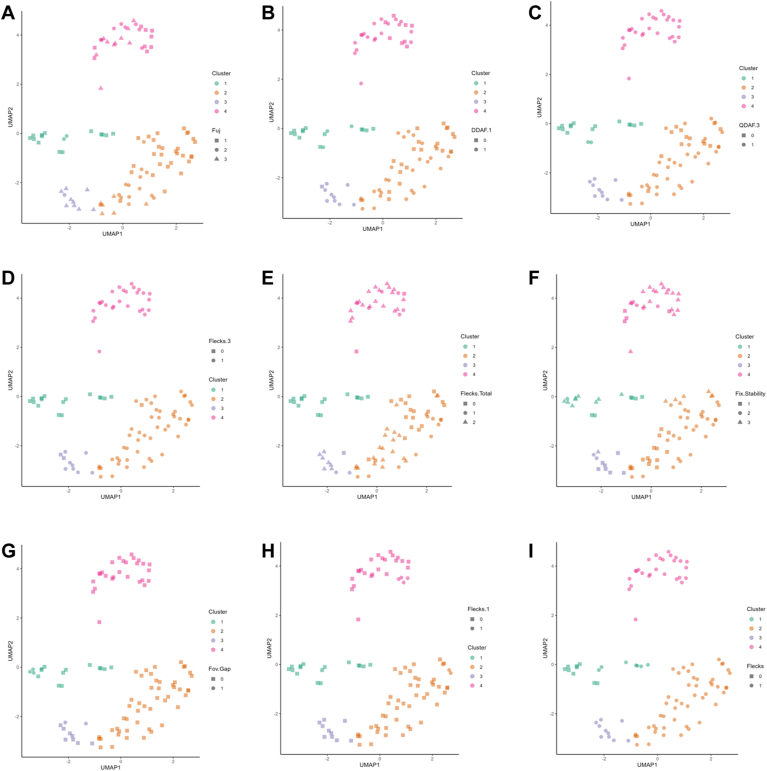
Uniform Manifold Approximation and Projection (UMAP) representation of categorical variables based on clusters. **A,** Fujinami score. **B,** presence of definitely decreased autofluorescence (DDAF) in zone 1. **C,** presence of questionably decreased autofluorescence (QDAF) in zone 3. **D,** presence of flecks in zone 3. **E,** total number of flecks in entire fundus autofluorescence. **F,** fixation stability. **G,** fovea gap. **H,** presence of flecks in zone 1. **I,** presence of flecks overall.

Figures 10 and 11 describe each phenotypic cluster based on the continuous and categorical variables, respectively, and highlight the major differences between each cluster. Quantitative descriptions of the continuous variables are found in Table 4. The remaining categorical variables may be found in Figure 12. Figures 13 and 14 illustrate the continuous and categorical variables using Uniform Manifold Approximation and Projection representation.

**Table 4:** Descriptive Statistics of Phenotypic Clusters of Patients with STGD and Pairwise Comparisons Between Clusters for Continuous Variables

Mod	Variables	Cluster 1	Cluster 2	Cluster 3	Cluster 4	*P* value					
		Mean (SD)	Mean (SD)	Mean (SD)	Mean (SD)	1 vs. 2	1 vs. 3	1 vs. 4	2 vs. 3	2 vs. 4	3 vs. 4
	VA	0.517 (0.466)	0.802 (0.317)	1.30 (0.504)	0.368 (0.506)	0.0430	0.0060	0.2100	0.0060	0.0003	0.0010
	Age	26.5 (13.3)	27.2 (12.4)	45.7 (12.3)	48.2 (15.4)	1.0000	0.0060	0.0002	0.0060	< 0.0001	1.0000
OCT											
	TMV	7.42 (0.627)	6.85 (0.894)	4.49 (1.22)	7.14 (1.01)	0.0830	0.0002	0.2160	0.0001	0.6060	0.0001
	CST	159.8 (45.0)	124.5 (31.6)	115.6 (23.8)	222.6 (50.6)	0.0270	0.0270	0.0060	0.3470	< 0.0001	< 0.0001
ERG											
	LA 30Hz A	65.4 (40.0)	61.2 (23.9)	25 (16.4)	48.9 (23.8)	0.7900	0.0110	0.4740	0.0004	0.1500	0.0260
	a-wave L	16.6 (2.75)	15.7 (1.83)	22 (3.59)	18.9 (2.95)	0.3470	0.0030	0.0100	< 0.0001	< 0.0001	0.0250
	b-wave L	45.4 (5.44)	43.6 (4.18)	46.6 (5.76)	51.2 (6.04)	0.4530	0.9570	0.0280	0.6200	< 0.0001	0.1740
	LA 30Hz L	27.6 (2.90)	29.4 (1.91)	34.1 (2.73)	30.6 (3.27)	0.0030	0.0010	0.0040	0.0002	0.1410	0.0160
MP											
	Fix.1	63 (32.2)	47.1 (25.4)	14.4 (12.8)	73.4 (27)	0.1370	0.0010	0.4820	0.0004	0.0005	< 0.0001

a-wave L = dark adapted 3.0 a-wave latency; b-wave L = dark adapted 3.0 b-wave latency; CST = central subfield thickness; ERG = electroretinogram; Fix.1 = percentage of fixation points 1 degree from the fovea; LA 30Hz A = light adapted 30Hz amplitude; LA 30Hz L = light adapted 30Hz Latency; Mod = modality; MP = microperimetry; SD = standard deviation; STGD = Stargardt disease; TMV = total macular volume; VA = logMAR visual acuity.

The first cluster included 16 out of 102 eyes (15.7%) from 9 unique subjects (7 bilateral symmetric eyes, 1 asymmetric eye, and 1 eye from a subject with data available only from a single eye). The mean age at the time of evaluation was 26.5 ± 13.3 years in this cluster. Mean VA was 0.517 ± 0.466 logMAR and was significantly better than VA in clusters 2 (confidence interval [CI] −0.592, −0.067; *P* = 0.043) and 3 (CI −1.20, −0.301; *P* = 0.006), but no different than that of cluster 4 (*P* = 0.21). Subjects in cluster 1 were significantly younger than those in cluster 3 (CI −29.7, −9.77; *P* = 0.006) and cluster 4 (CI −31.9, −13.1; *P* = 0.00020) but similar to those in cluster 2 (*P* > 0.05). On OCT imaging, TMV of eyes in cluster 1 was similar to that of eyes in cluster 2 (*P* = 0.083) and cluster 4 (*P* = 0.216), all of which were greater than TMV in cluster 3. Central subfield thickness of eyes in cluster 1 was greater than that of cluster 2 (CI 11, 62; *P* = 0.027) and cluster 3 (CI 5, 80; *P* = 0.027) but less than cluster 4 (CI −96, −25; *P* = 0.006). The mean DA 3.0 a-wave latency was significantly lower than eyes in cluster 3 (CI −8, −2; *P* = 0.003) and cluster 4 (−5, −1; *P* = 0.01) but similar to that of cluster 2 (*P* = 0.347). In ERG testing, eyes in cluster 1 additionally had the lowest LA 30Hz latency values of all 4 clusters. Eyes in cluster 1 predominantly had 1 atrophic lesion with a homogeneous background (14 eyes, 87.5%), no DDAF in zone 1 (13 eyes, 81.3%), no QDAF in zone 3 (14 eyes, 87.5%), no flecks in the entire FAF imaging (9 eyes, 56.3%), and stable fixation (9 eyes, 56.3%).

The second cluster included 49 eyes (48.0%) from 27 unique subjects (22 bilateral symmetric eyes and 5 asymmetric eyes) with a mean VA of 0.801 ± 0.317 logMAR, which was significantly worse than VA of eyes in clusters 1 (CI −0.59, −0.067; *P* = 0.043) and 4 (CI 0.398, 0.699; *P* = 0.00025), but greater than that of cluster 3 (CI −0.854, −0.204; *P* = 0.006). Similar to cluster 1, subjects in this cluster were younger (mean 27.2 ± 12.4) than those in cluster 3 (CI −27.4, −9.75; *P* = 0.006) and cluster 4 (CI −27.9, −13.8; *P* < 0.0001). On OCT imaging, eyes in cluster 1 and 2 had comparable mean TMV values (*P* = 0.083). Central subfield thickness of eyes in cluster 2 were significantly less than those of clusters 1 (95% CI 11, 62; *P* = 0.027) and cluster 4 (CI −120, −71; *P* < 0.0001). In ERG testing, the mean DA 3.0 a-wave latency was significantly lower than in eyes in cluster 3 (CI −8, −5; *P* < 0.0001) and cluster 4 (CI −5, −1; *P* < 0.0001), but similar to that of cluster 1 (*P* = 0.347). Light adapted 30Hz latency was greater than eyes in cluster 1 (CI −3, −1; *P* = 0.003) but less than that of cluster 3 (CI −7, −3; *P* = 0.001). The FAF imaging of most eyes in cluster 2 was characterized by 1 atrophic lesion with homogeneous background (29 eyes, 59.1%), DDAF present in zone 1 (29 eyes, 59.1%), QDAF present in zone 3 (36 eyes, 73.5%), flecks in zone 3 (48 eyes, 98%), and > 100 flecks overall (20 eyes, 40.8%). Most eyes in cluster 2 had relatively unstable fixation (27 eyes, 55.1%). Cluster 2 additionally had the highest percentage of eyes with relatively unstable fixation compared to all other clusters.

The third cluster included 10 eyes (9.8%) from 6 unique subjects (4 bilateral symmetric eyes and 2 asymmetric eyes) with an average VA of 1.30 ± 0.504 logMAR, which was significantly worse than all other clusters. Subjects in cluster 3 (mean 45.7 ± 12.3) were significantly older than eyes in both clusters 1 (−29.7, −9.77; *P* = 0.006) and 2 (CI −27.4, −9.75; *P* = 0.006). On OCT imaging, cluster 3 had eyes with the lowest TMV of all 4 clusters and a CST lower than that of eyes in cluster 1 (CI 5, 80; *P* = 0.027) and cluster 4 (CI −96, −25; *P* < 0.0001). In ERG testing, this cluster had eyes with the lowest LA 30Hz amplitude and percentage of fixation points 1 degree from the fovea but the highest LA 30Hz latency and DA 3.0 a-wave latency compared to eyes in all other clusters. Eyes in cluster 3 predominantly had multiple atrophic lesions with a heterogeneous background (10 eyes, 100%), DDAF present in zone 1 (9 eyes, 90%), QDAF present in zone 3 (10 eyes, 100%), flecks present in zone 3 (8 eyes, 80%), greater than 100 flecks overall (7 eyes, 70%), and unstable fixation (7 eyes, 70%). Compared to all other clusters, eyes in cluster 3 had the highest percentage of eyes with multiple atrophic lesions and a heterogeneous background (100%), DDAF present in zone 1 (90%), and unstable fixation (70%).

**Figure 15:**
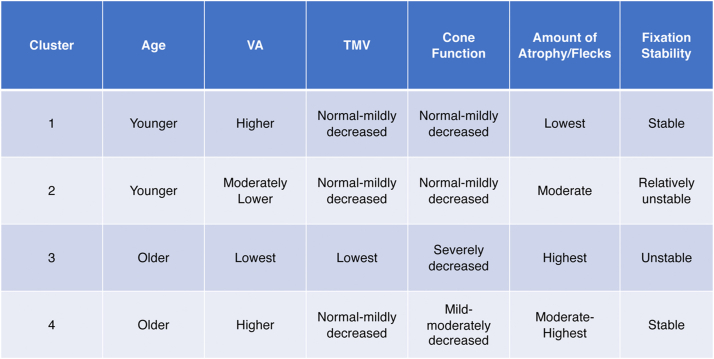
Phenotypic summary of the 4 clusters of Stargardt disease identified within the study sample. TMV = total macular volume; VA = visual acuity.

Finally, the fourth cluster comprised 27 eyes (26.5%) from 16 unique subjects (11 bilateral symmetric eyes, 4 asymmetric eyes, 1 eye from a subject with data available only from a single eye) with an average VA of 0.368 ± 0.506 logMAR, which was significantly better than VA from eyes in cluster 2 (CI 0.398, 0.699; *P* = 0.00026) and cluster 3 (CI 0.671, 1.40; *P* = 0.00095). Subjects in cluster 4 were significantly older than subjects in clusters 1 (CI −31.9, −13.1; *P* = 0.00020) and 2 (CI −27.9, −13.8; *P* < 0.0001). On OCT imaging, CST was highest in cluster 4 out of all clusters, and the average number of fixation points 1 degree from the fovea was greater than in both clusters 2 (CI −44, −16; *P* = 0.00053) and 3 (CI −82, −42; *P* < 0.0001). In ERG testing, DA 3.0 a- and b-wave latencies in cluster 4 were both significantly greater than clusters 1 (a-wave: CI −5, −1; *P* = 0.01; b-wave: CI −9, −2; *P* = 0.028) and 2 (a-wave: CI −5, −1; *P* < 0.0001; b-wave: CI −10, −5; *P* < 0.0001). On FAF imaging, eyes in cluster 4 predominantly had multiple atrophic lesions with heterogeneous backgrounds (10 eyes, 37%), DDAF present in zone 1 (19 eyes, 70.4%), QDAF present in zone 3 (27 eyes, 100%), flecks present in zone 3 (27 eyes, 100%), greater than 100 flecks overall (19 eyes, 70.4%), and stable fixation (17 eyes, 63%). Compared to all other clusters, eyes in cluster 4 had the highest percentage of eyes with flecks in zone 3 and stable fixation. Furthermore, cluster 4 had the most equal distribution of eyes with different background heterogeneities and number of atrophic lesions (33% of eyes with 1 atrophic lesion and a homogeneous background, 30% with 1 atrophic lesion and a heterogeneous background, and 37% with multiple atrophic lesions and a heterogeneous background). A summary table of all 4 clusters and their phenotypic features may be found in Figure 15.

### Related Subjects

Six subjects were found to be biologically related to other subjects in the study: 3 unique sibling pairs were identified. The subjects in 2 of the 3 pairs belonged to different clusters (clusters 1 and 2), while the subjects in the third pair belonged to the same cluster (cluster 1).

## Discussion

This study identified 4 clusters of STGD by clustering imaging (FAF and OCT) and retinal functional test (ERG and MP) data through unsupervised algorithms. The distinct profiles were defined as (1) younger subjects with better VA, normal-mildly decreased macular volume, normal-mildly decreased cone function, low levels of atrophy and flecks, stable fixation, and a small percentage of eyes with fovea gaps; (2) younger subjects with moderate VA, macular volume similar to clusters 1 and 4, normal-mildly decreased cone function, moderate levels of atrophy and flecks, and relatively unstable fixation; (3) older subjects with the lowest VA, lowest macular volume, high levels of cone dysfunction, atrophy, and flecks, unstable fixation, and a small percentage of eyes with fovea gaps; and (4) older subjects with higher VA, normal-mild macular volume, mild-moderately decreased cone function, high levels of atrophy and flecks, and stable fixation. Our results provide a framework to develop a novel classification system that considers the complexity of the STGD phenotype by incorporating multiple structural and functional markers including objective and psychophysical test data. With further validation, such a classification approach may be used to stratify patients for diagnostic, prognostic, or therapeutic purposes.

Previous studies have proposed STGD classifications, though they have typically only used a few imaging or retinal function testing modalities.^20^^,^^24^, ^25^, ^26^^,^^33^, ^34^, ^35^ Fishman et al. proposed that STGD disease progression may be identified through 4 stages based on FAF and ERG data.^6^^,^^36^ Lois et al.^24^ identified 3 groups of subjects based on ERG data that represented distinct STGD phenotypes rather than separate stages of progression: (1) pattern ERG abnormalities with normal rod and full-field ERG, (2) normal rod ERG with decreased cone DA 3.0 b-wave amplitudes, and (3) loss of both cone and rod function. Fujinami et al.^25^ were one of the few that classified subjects using multiple imaging and retinal function tests, yet machine learning was not incorporated to verify the results. Fujinami et al.^25^ grouped subjects based on disease severity as established by the appearance of the fundus, autofluorescence pattern, VA, ERG pattern, and age at onset. While this classification method provides invaluable knowledge on phenotypic diversity of STGD, these phenotypes are not statistically verified. Microperimetry was also not included in this study, and findings from each imaging modality were summarized into only 1 score. Our study builds on this prior work by incorporating comprehensive retinal function testing and extracting multiple variables from each imaging and retinal function modality to assess which elements explain the most variance in the data. Furthermore, this work uses correlation analysis, FAMD, and unsupervised clustering to assess which variables in STGD are correlated between different imaging/testing modalities.

With regards to correlations between variables, the Fujinami classification was positively correlated with background heterogeneity, atrophy beyond the arcades, and the presence of DDAF in zone 3. The Fujinami classification takes into consideration the number of atrophic lesions in the macula. Atrophy in STGD has been documented to spread in a centrifugal manner. Therefore, it would be expected for eyes with a higher Fujinami score (more atrophic lesions) to have DDAF present in zone 3 as well as atrophy beyond the arcades. Eyes with higher Fujinami scores similarly tended to have flecks beyond the arcade in all clusters. This is in line with previous studies that report on fleck and atrophic lesion progression occurring in a centrifugal manner.^37^ The correlation between presence of DDAF in zone 1 and in zone 2 may be attributed to the centrifugal manner in which atrophic lesions grow, with lesions in zone 1 extending to zone 2 over time. The presence of flecks was further associated with presence of QDAF in zone 2.

Visual acuity of eyes in all 4 clusters was significantly different, except for between clusters 1 and 4. However, VA was not 1 of the 36 variables identified in the first FAMD analysis. Therefore, though the clusters differed in their VA measurements, it is difficult to conclude that VA can be a variable used to categorize patients with STGD. Visual acuity is ultimately a multifactorial measurement and may vary based on conditions including refractive error, background brightness, and operator variability. These factors may have increased variability of VA in comparison to the other variables measured.

Flecks are one of the most characteristic features of STGD. They are a result of accumulation of lipofuscin in the RPE due to mutated *ABCA4* transport proteins and are best visualized on FAF.^4^, ^5^, ^6^ Prior studies suggest that flecks present in the periphery of the macula and a greater number of flecks is suggestive of an advanced stage of the disease.^37^^,^^38^ Clinically, macular areas with high fleck density are correlated with poor retinal sensitivity as measured by MP^39^ and degeneration of photoreceptor cells.^40^ We found that clusters 2, 3, and 4 had similar distributions of eyes with flecks in zone 3, yet varied in a number of FAF, OCT, ERG, and MP findings. Since the centrifugal spread of flecks in FAF may be a proxy of disease stage, these clusters may represent different phenotypes rather than different stages of disease because they demonstrate similar fleck patterns but vary in other parameters. The clustering among the sibling pairs further suggests that these clusters may represent unique phenotypes. Three biological sibling pairs were identified, and the subjects of 1 sibling pair belonged to separate clusters. These clusters did not significantly differ in age, meaning that the differences in this sibling pair's disease were likely due to phenotypic differences as opposed to different stages of disease progression.

Cluster 1 appeared to be the mildest of the 4 identified phenotypes: the total number of flecks in cluster 1 eyes was significantly different compared to those in all other clusters, with cluster 1 predominantly having eyes with no flecks or < 50 flecks. Similarly, eyes in cluster 1 had the least atrophy.

Cluster 3 had the lowest macular thickness values, and subjects in this cluster were significantly older than those in clusters 1 and 2. Cluster 3 may represent the most severe phenotype identified in this study given these findings of macular thickness in conjunction with poor cone function and fixation stability and high levels of atrophy. Cluster 3 also appeared to have significantly worse cone function than the other clusters. Regarding MP data, eyes in cluster 3 had a dramatically lower percentage of fixation points 1 degree from the fovea in comparison to the other clusters. This was expected, as this cluster additionally had the highest percentage of eyes with unstable fixation.

There are several limitations of this study. Firstly, our relatively small sample size limits the generalizability of our findings and the ability to validate the clusters. Secondly, the age of onset and disease progression were both not recorded in this study. This limits the ability to conclude whether we have identified different phenotypes of diseases or different stages in the disease process. Finally, changes in imaging technologies over time and operator variation may have impacted data acquisition. However, all tests were performed with the most up to date software version at the time and we envision that future studies will harmonize the allowable software versions.

## Conclusion

Phenomapping of STGD, incorporating multimodal structural and functional features, may be a useful tool to categorize patients based on phenotype. Our results demonstrate that the phenotype clusters differ based on several variables, indicating that the phenomap may be further simplified for implementation in community care settings, thus facilitating research and clinical care in that context. Further studies could focus on discerning whether the phenomap clusters represent fundamental allelic differences, or alternatively, different stages of disease evolution. Phenomapping STGD may have potential utility for prognostication and treatment assignment with gene, cell, or pharmacologic therapy modalities, although this prediction remains to be validated in the future interventional settings.
